# Supplementary material for: Protocol for a longitudinal study investigating the role of anxiety on academic outcomes in children on the autism spectrum
Source: PLoS One. 2021 Sep 16;16(9):e0257223. doi: 10.1371/journal.pone.0257223 (PMC8445440; doi:10.1371/journal.pone.0257223)
Supplement: S1 File — (PDF) [file pone.0257223.s002.pdf]

1. Title

"Cool for School": Can a parent-group help to reduce anxiety in children with autism?

2. Project Team Roles & Responsibilities

Dr Dawn Adams
Senior Lecturer, Autism Centre of Excellence, Griffith University.

Dr Adams is a senior lecturer in autism and a certified clinical psychologist with expertise in child and parental mental health. She has published extensively in the area of anxiety and autism over the past four years and is Lead CI on the ARC Linkage grant. Dr Adams will oversee the project and staff, the project budget, and coordinate the quantitative analysis and interpretation of the data, and management of the database for the project and reporting of results.

Dr Kate Simpson
Lecturer, Autism Centre of Excellence, Griffith University.

Dr Simpson is a lecturer in autism and a qualified occupational therapist and teacher. Her research focuses on how characteristics of autism influence participation and engagement across a range of environments. She will be responsible for training and oversight of the Research Assistant. She will contribute to data collection, analysis, and reporting of results.

Dr Stephanie Malone
Postdoctoral Research Fellow, Autism Centre of Excellence, Griffith University.

Dr Malone is a postdoctoral research fellow with experience in the development of children's early cognitive and academic skills. Dr Malone will have responsibility for the day-to-day management of the project to ensure efficient and effective coordination of activities. She will also assist with the recruitment of participants, participant assessments, recruitment and training of clinicians to implement the modified CLK program, data entry, analysis and reporting of results. She will also be the principal family liaison contact person and be responsible for ethical clearances and confidential storage of client records.

Dr Madonna Tucker
Research Manager, AEIOU Foundation.

Dr Tucker holds a key position within AEIOU Foundation, leading the organisation's program of research. She also has oversight of the child assessment and reporting program for the organisation. As such she has extensive experience liaising with families. For this project, she will be responsible for parent liaison, disseminating information to parents about recruitment, and answering questions from parents about the project.

Prof Ronald Rapee
ARC Laureate Fellow and Distinguished Profressor, Department of Psychology

Prof Rapee is a distinguished professor and ARC Laureate at Macquarie University. His research expertise centres on anxiety and related disorders across the lifespan, best known for theoretical models of development of anxiety disorders and creation of Cool Kids, Cool Kids for Autism and Cool Little Kids. He will provide knowledge and expertise in the CLK program and

guide modifications to the program with PI Rodgers and CI Adams. He will advise on training for intervention implementation, study design, and data analysis, and will contribute to reporting of results.

Dr Jacqui Rodgers
Senior Lecturer, University of Newcastle upon Tyne.

Dr Rodgers is a senior lecturer and autism researcher in the Institute of Neuroscience. She leads a programme of work that aims to advance the conceptualisation, assessment and treatment of anxiety in autism. Dr Rodgers will make modifications to the CLK program to incorporate material on intolerance of uncertainty to better target the CLK program for parents who have a child with autism.

3. Resources

This study consists of four stages: initial assessment (stage 1), intervention (stage 2), short-term follow-up assessment (stage 3), and one-year follow-up assessment (stage 4). At Stages 1, 3, and 4, data will be captured from the participating parent(s) and their child.

Direct child assessments focus on gaining an understanding of the child's autism severity and cognitive ability. A trained member of the research team will administer these assessments within a 1:1 setting. At Stage 4, additional assessments will be included to address school-related skills.

Parent completed assessments consist of a series of standardised questionnaires made available online via REDCap Survey.

Note. Three of these assessments are administered by our partner organisation (AEIOU) as part of their existing intake assessments (marked with an asterisk below). As such, these will not be administered at Stage 1 for those participants recruited via AEIOU.

Direct Child Assessments

Autism severity: The *Autism Diagnostic Observation Schedule (ADOS-2)** will be used to measure autism severity. Trained members of our research team will administer this at Stage 1, 3 and 4. Although originally designed as a diagnostic assessment, the ADOS-2 is commonly used in research as a quantitative measurement of autism severity with good reliability and validity. It is considered the gold standard assessment of autism.

The ADOS-2 consists of a series of social activities (or 'presses') designed to elicit behaviours directly relevant to the diagnosis of autism. There are five modules, each tailored to a specific developmental level and/or chronological age. The most appropriate module will be selected on a child-by-child basis. Given the age of the children (4- to 5-years), this is likely to be module 1 or 2. Each module can be administered in 40- to 60-minutes. Social activities included in this assessment consist of structured and unstructured activities, such as make-believe play using a series of age-appropriate items, responsiveness to social prompts (e.g. name, eye gaze and pointed), and

construction puzzles. As per the ADOS-2 manual, a parent will accompany children completing Module 1 or 2.

When coding and scoring the ADOS-2, it is useful to have access to video footage of the assessment. As such, parents will be asked to provide consent for their child to be video-recorded while completing the ADOS-2.

Cognitive ability: The *Mullen Scales of Early Learning (MSEL)** is a well-established, standardised measure of ability for children age 0-68 months. This will be used to measure child ability (visual reception, receptive language, expressive language, fine motor and gross motor) and provide a Developmental Quotient (DQ). The reliability and validity of the MSEL has been assessed in young children with autism and is commonly used as both a descriptive and outcome measure in this population. Trained members of our research team will administer this assessment at Stage 1, 3 and 4.

School-related skills: We are developing a series of measures to assess children's school-related abilities. These focus on abilities such as school connectedness, motivation, engagement, study skills, socialisation and academic self-concept. These tasks will be developed during 2020. Once finalised, a modification form will be submitted for the inclusion of these measures at Stage 4 of the current study.

Parent Completed Questionnaires

- Preschool Anxiety Scale Revised (PAS-R)
- Anxiety Scale for Children with Autism Spectrum Disorder (ASC-ASD)
- Intolerance of Uncertainty Scale (IUS-P)
- Vineland Adaptive Behaviour Scales 3rd Edition (VABS-3)*
- Repetitive Behaviour Scale – Early Childhood (RBS-EC)
- Depression Anxiety Stress Scales (DASS)
- Children's Anxiety Life Interference Scale (CALIS)
- Responses to Uncertainty and Low Environmental Structure questionnaire (RULES)
- Social Communication Questionnaire (SCQ; a measure of autism symptoms)
- Short Sensory Profile II (SSPII)
- As with the direct child assessments, we will develop a series of questionnaires to assess parents' perceptions of their child's school-related skills (e.g., motivation, engagement, school connectedness, study skills, socialisation and academic self-concept). These questionnaires will be submitted as a modification and, subject to ethical approval, will be implemented during Stage 4 of the study in 2021.

Additional Resources

- Clinician and parent manuals for the Cool Little Kids (modified for Autism) program. This modified version has been piloted and was found to be acceptable by the participating parents (GU Ref No: 2019/624).

The funding source for this project is ARC LP180100318

4. Background

Research Program Structure: This study forms part of a nested program. The overarching aim is to investigate how anxiety, a commonly occurring condition in autism, impacts attitudes and behaviours that facilitate students' participation in, and ability to benefit from, academic instruction in the classroom. The current study forms the **first phase** of this nested program.

As outlined in the successful Linkage Project grant application, the current study will compare the anxiety levels of two groups of 4- to 5-year-old children with autism: those whose parents have completed the modified Cool Little Kids (CLK) intervention and those that have not. This will likely result in an overall group of children who have a range of anxiety levels; this is of interest in phase two (see below). The randomised control trial method used to allocate parents to the intervention and control groups will allow us to address a secondary aim: Is the modified CLK program able to reduce/prevent anxiety in children with autism? Although a larger scale RCT would be necessary to confidently demonstrate efficacy, the current study involving 64 families will provide important insights into the possible benefits of the intervention.

Ethical approval for the **second phase** of this study will be sought in 2020 from Griffith University and the necessary governing bodies (e.g. Brisbane Catholic Education, Education QLD). Parents who participated in phase one will be invited to take part in phase two. Data collected in phase two will include the teacher's perspective on the child's academic functioning (e.g. reading, mathematics and critical thinking) and academic enablers (e.g. motivation, engagement, interpersonal skills and study skills) within the first 6-months of formal schooling. By combining the data from phase one and two, we can determine whether moderating anxiety in children with autism is associated with academic enablers. This will allow our overarching research questions to be addressed:

1. Is there evidence that anxiety is associated with academic enablers in children with autism?
2. Does anxiety mediate the relationship between autism and academic enablers in children with autism?

Background Literature: Anxiety disorders are the most common form of mental disorder in young people, affecting around 7% of Australian youth (Lawrence et al., 2015). These disorders are even more common in children with autism, affecting up to 40% of the population (van Steensel & Heeman, 2017) with an even larger number showing sub-clinical levels of elevated anxiety (Vasa et al., 2013).

Our latest research suggests that children with autism may begin to experience the early signs of anxiety before they even start school. An extensive literature has demonstrated the efficacy of skills-based intervention programs to reduce heightened anxiety among children as young as 3 years of age (Mychailyszyn, 2017). One such program is Cool Little Kids (CLK), which has demonstrated excellent outcomes in the reduction of anxiety among preschool-aged children. Parallel research has shown similar reductions in anxiety among children who are comorbid for anxiety and autism (Mychailyszyn, 2017; Ung et al., 2015). The core treatment strategy in the CLK program is in vivo exposure, which primarily works via

extinction to shift expectations of threat (Craske et al., 2008). In a recent analysis, CLK was found to lead to reductions in anxiety among a group of preschool-aged children with comorbid autism (Craske et al., 2008). Although CLK has shown promise in reducing anxiety among young children with autism, its processes focus primarily on one mechanism of anxiety, threat expectancy. In addition to heightened threat expectancy, an additional key mechanism in the maintenance of anxiety is a reduced ability to tolerate uncertainty (intolerance of uncertainty (IU); Carleton et al., 2010). High levels of IU are likely to be especially pertinent to children with autism. More complete reduction in anxiety might therefore be achieved by incorporating processes that reduce IU, given its key role in the expression of anxiety among children with autism. Modifying the CLK program by incorporating processes to reduce IU should thus allow greater reduction in anxiety among children with autism.

We are currently piloting such a modified version of the program with a small group of parents of children with autism (*n* = 4; GU Ref No: 2019/624). The parents have reported positive feedback on this program during the intervention sessions. The current study aims to determine whether the intervention is able to assist in reducing children's anxiety levels= (as planned as part of the ARC Linkage project to investigate the role of anxiety in the successful development of academic enablers in children with autism). The implementation of this trial will allow the following questions to be addressed:

1. Does the modified CLK program lead to a reduction in the levels of anxiety experienced by young children with autism?
2. Is participation in the modified CLK program associated with lower levels of anxiety post-intervention, compared to children in a control group (i.e. whose parents did not complete the program)?

Drawing upon the initial findings of the CLK and CUES programs, it is hypothesised that parent participation in the modified CLK program will be associated with a reduction in children's anxiety levels from the initial assessments to the follow-up assessments. It is further predicted that children whose parents participated in the modified CLK program will demonstrate lower levels of anxiety post-intervention, than those children in the control condition (i.e., whose parents did not participate in the program). The primary outcome of this study is to provide preliminary evidence regarding whether the modified CLK program can reduce (or prevent) anxiety in children with autism. It will also establish children's anxiety levels in pre-school which will be informative in phase two (should parents consent to their continuing participation).

5. Project Description

Setting

The intervention will be conducted at Griffith University campuses across Queensland or AEIOU Foundation centres (Gold Coast, Logan, Nathan, Camira, Bray Park, Sunshine Coast, and Toowoomba). These locations have training/meeting rooms ideal for the purpose. Parents will be invited to attend the research sessions at a geographically convenient AEIOU or Griffith University location. Although AEIOU facilities may be used for the intervention, no AEIOU personnel will be present or involved in running the intervention.

Methodological Approach

We will aim to recruit 64 parents of young children with autism (aged 4- to 5-years-old) through social media (Griffith University Autism Centre of Excellence Facebook page) and early intervention centres operated by our research partner, AEIOU Foundation. AEIOU centres are accessed by up to 280 children annually.

Following a randomised control trial (RCT) method, parents will be randomly allocated to one of two conditions: intervention (whereby parents will participate in all stages of the study, including the modified CLK program) and control (in which parents will complete the initial and both follow-up assessment points but not engage in the CLK program). This design allows comparisons to be made between the two groups, which will assist in determining whether the intervention was successful in reducing (or preventing) anxiety in young children with autism, or whether any reduction (or maintenance) simply reflects a natural change due to general development.

Two alternative procedures were considered, yet were both dismissed due to methodological limitations:

1) Wait list control: The modified CLK program is aimed at young children (3- to 6-years; Rapee, Kennedy, Ingram, Edwards, & Sweeney, 2005), thus upon completion of the study the participating children would exceed the target age group for the intervention.
2) Offering an alternative intervention to the control group: This technique makes it difficult to determine if any differences between the two groups observed post-intervention is a result of the CLK program or the alternative intervention completed by the control group.

It is worth noting that this is the first trial of this modified CLK intervention, so its efficacy as an early intervention for anxiety in autism is unknown. Using this RCT design (i.e. intervention vs. control) allows us to be relatively certain that any post-intervention differences in anxiety level between the two groups are a result of the intervention itself. This type of clear-cut information is vital, as it will provide preliminary evidence of the efficacy of this program, and therefore whether it would be beneficial for future research to conduct a full-scale trial into its utility.

To determine group allocation, each participating family on the database will be allocated a random number via a random number generator. This will then be used to evenly distribute the participants across the two conditions, with those assigned an odd number placed in the intervention group (n = 32), and even numbers placed in the control group (n = 32).

All participants, regardless of their group allocation, will complete the measures listed in Section 3 at Stage 1 (initial assessments), Stage 3 (short-term follow-up) and Stage 4 (one-year follow-up). The longitudinal nature of this study allows for us to determine the short-term and longer-term effects of the CLK program on the anxiety levels of children with autism. We are also able to examine whether participation in this intervention had any impact on the more distal constructs of, for example, maternal stress (DASS).

Participants will be recruited and assessed in three waves:

1. Beginning January 2020: Recruitment will focus on social media
2. Beginning February 2020: Recruitment via social media and AEIOU
3. Beginning June 2020: Recruitment via social media and AEIOU

For each wave, participation will begin with the completion of the initial assessments (see Section 3), followed by the modified CLK intervention for those in the intervention group (administered as per the manual), short-term follow-up assessments (within two months of CLK program completion) and long-term follow-up assessments (one-year later). The initial three stages of the study will occur over a 5- to 6-month time frame, with the final follow-up assessments occurring one year later.

Participant Recruitment

Recruitment through AEIOU: We will first submit for ethical approval of the research project through AEIOU. Once approved, the AEIOU Research Manager (Dr Tucker) will identify parents of children attending AEIOU centres who meet the criteria outlined above (i.e. aged 4- to 5-years old). Parents identified as meeting these inclusion criteria will be provided with a letter of invitation, an information sheet and consent form (all attached) to inform them of the study.

Social Media: An invitation to participate in this research study will be made available on our social media page (Autism Centre of Excellence Griffith University Facebook page). The text will read as follows:

> Are you a parent of a child who will be starting school in 2021 in South East Queensland? We're looking for parents to take part in a trial of a 6-session parent intervention to reduce (or prevent) anxiety in children with autism. For more information, email coolforschool@griffith.edu.au.

Participants

64 parents of children with autism (aged 4- to 5-years). The sample size is deemed appropriate for this type of study design and is considered large enough to identify any significant differences between the two groups (intervention vs. control).

Confirmation of autism diagnosis: All children in AEIOU centres have an autism diagnostic assessment measure undertaken at intake (the ADOS; Autism Diagnostic Observation Schedule); children will only enter into this study if they meet the autism cut-off on this measure. If they are recruited through social media, we will conduct the ADOS at Stage 1 to confirm diagnosis and only offer the intervention if children meet the diagnostic cut-off on this measure.

Recruitment strategy:

AEIOU: The intervention centre will send information about the study to families who meet the eligibility criteria. This information will be sent via two means, firstly an email from the AEIOU Communications division and secondly a printed copy will be available via the child's centre. If needed, member(s) of the research team will offer drop-in sessions for the invited parents to ask any questions about the research.

Parents that wish to participate will complete the consent form, put it inside a sealed envelope provided with the consent materials, and then return it to the child's early intervention centre within 14 days of receiving the invitation. The Centre Manager will notify their research manager and/or the research team when envelopes have been received so that the contact information can be shared with the research team.

Social Media: An advert will be posted on our social media page (Autism Centre of Excellence Griffith University Facebook page) inviting parents of children with autism aged 4- to 5-years to participate. Parents will be asked to indicate their interest by sending an email to our project specific email address. We will then reply to this with the information and consent materials. If requested, we will also post a hard copy of this material. If needed, member(s) of the research team will offer drop-in sessions for the invited parents to ask any questions about the research.

Parents who wish to participate can complete an online consent form or print a PDF and email it or return the consent form via email or post within 14 days of receiving the invitation (date stamp on envelope used to indicate date of response). If parents choose to respond via the online consent form or email, they will be asked to complete a hard copy of the consent form when first meeting with a member of our research team (e.g. when attending the child-directed assessments).

Incentive: Parents will be provided with a gift card after their completion of each assessment stage. The value of the gift card will increase as they progress through the study: $25 for completion of Stage 1, $50 for Stage 3, and $100 for Stage 4. This increase in value reflects their continued commitment to the project. It also acts as an incentive for parents to complete each of the assessment stages; this is vital as in order to determine the efficacy of the intervention, we need as many parents as possible to complete the project.

We will also provide parents with confidential research reports; one after Stage 1 (for those recruited through social media only) and after Stages 3 and Stage 4 for all participants). These will detail their child’s level of IQ and adaptive functioning and can be shared with anyone they wish as it may assist in providing others with further information on their child’s ability and skills.

Sample management: Parents who consent to participate based on the first social-media advert will be allocated to wave one (until this reaches capacity), with any additional parents allocated to wave two and three as needed. For wave two, invitations will be circulated to AEIOU parents (and social media if necessary). In the unlikely event that all invitations are accepted, these families will be divided across wave two and three. If only a subsample accepts the invitation, they will be allocated to wave two (until this reaches capacity), with any additional parents allocated to wave three. The AEIOU uptake rate observed here will give us a better idea of the number of potential families to circulate the study information to for the third wave.

Any parents who accept the invitation in the third round of recruitment will be allocated to wave three.

Given our staged recruitment strategy across multiple waves, it is unlikely that a large number of additional parents will wish to participate. The number of parents approached at each wave can be adjusted to reflect the number of parents who have already consented to the project. We anticipate that the three waves may not be equal in size and this has been factored into the study design and therapist availability to run groups.

Procedure after consent is given

Upon obtaining consent, the postdoctoral research fellow / project manager (Dr Malone) will contact consenting parents and provide a link to the online questionnaires. Parents who complete the initial assessments will be advised of their randomly allocated group membership (intervention vs. control). Those in the intervention group will be asked to select a parent group at a convenient location and times for them. All parents will be reminded of the contact details for the research team.

Although parents have provided their informed consent to participate in the research, their children will be asked to provide their assent (where possible) prior to completing the direct child assessments. Given that the verbal ability of some children may be relatively low, their vocalisations and physical behaviours will be taken into consideration when determining their assent. The child's parent(s) will also be present during these assessments, so can assist in interpreting the child's behaviours. At all times, the child's verbal and non-verbal behaviours will be observed. If at any point either the researcher or child's parent feel that the child would like to stop or have a break, the researcher will respond accordingly by either discontinuing or pausing the assessment. Additionally, children with autism often communicate using picture communication symbols. Where appropriate, we will make a "break" symbol available, so the child is able to pause/stop the assessments by presenting this symbol.

Research Activities

Participants in the intervention group will attend a parent-mediated, group-based intervention designed to prevent/reduce anxiety in their children with autism. The intervention consists of six two-hour sessions spaced over a school term. The intervention has a clinician manual and a parent workbook and parents are asked to undertake some homework activities between sessions.

Parents and children will also participate in initial and follow-up assessments (stage 1, 3 and 4) that will take place approximately two weeks prior to the intervention, two weeks following the intervention, and one-year later. Children recruited via AEIOU will only require the follow-up assessments (stage 3 and 4) as they will have already completed the ADOS and MSEL during their AEIOU intake assessment. All stage 1, 2 and 3 activities will be completed by December 2020; the final follow-up will be conducted the following year (2021).

Data Collection/Gathering

Parents: All parents will be asked to complete questionnaires at Stage 1, 3, and 4 (initial assessments, short-term follow-up and one-year follow-up). These questionnaires will be administered using REDCap so they can be completed online. The questionnaires ask parents to report on their child's anxiety, intolerance of uncertainty, repetitive behaviours, the impact anxiety may have on daily life, and other relevant constructs. Note that at Stage 1, parents recruited via AEIOU will not have to complete the Vineland caregiver questionnaire as this is administered as part of the AEIOU intake procedure.

At Stage 4, additional parent questionnaires will be added to assess their child's school-related skills (subject to approval of modification request; see Section 3). All parents will be invited to answer these items. We will provide parents with additional details regarding Stage 4 assessments closer to the time.

The standardised questionnaires completed by the parents are as follows:

- Preschool Anxiety Scale Revised (PAS-R)
- Anxiety Scale for Children with Autism Spectrum Disorder (ASC-ASD)
- Intolerance of Uncertainty Scale (IUS-P)
- Vineland Adaptive Behaviour Scales 3rd Edition (VABS-3)*
- Repetitive Behaviour Scale – Early Childhood (RBS-EC)
- Depression Anxiety Stress Scales (DASS)
- Children's Anxiety Life Interference Scale (CALIS)
- Responses to Uncertainty and Low Environmental Structure questionnaire (RULES)
- Short Sensory Profile II
- Social Communication Questionnaire (SCQ; a measure of autism symptoms)

In addition, parents recruited via an early-intervention centre will be asked to consent to the sharing of assessment data already collected by their child's AEIOU Foundation centre. Sharing of these data will be beneficial in describing the participant group and will save repeating these assessments unnecessarily.

Specifically, the following assessment data will be requested:

- Autism Diagnostic Observation Scale-Second Edition
- Mullen Early Learning Scales
- Vineland Adaptive Behaviour Scale- Third Edition

Records will be kept of parent attendance at group intervention sessions and homework completion. A trained member of the research team, using a checklist designed for this purpose, will monitor fidelity with the clinician manual while implementing the intervention. To further assist with monitoring fidelity, we will also ask parents to provide their consent for the sessions to be audio recorded. This will allow the research team to ascertain if the sessions are being conducted as per the intervention manual. Any resulting audio recordings will be stored securely on a Griffith University server, and will be accessed only by members of the research team.

It is possible that through completing questionnaires about their child's anxiety and its impact, parents may experience some distress as they reflect on their child's abilities and challenges. Further, as parents are working through the intervention and trying strategies with their child, there may be changes in the child's behaviour that could impact on them and their family. Parents will be advised during the intervention sessions to speak to the project team or their healthcare professional if they experience these concerns.

Children: At Stages 1, 3, and 4, children will be asked to complete a series of assessments measuring autism severity, cognitive ability and (at Stage 4) school-related skills. Any children recruited via AEIOU will only need to complete the assessments at Stage 3 and 4, as assessments of autism severity and cognitive ability are completed as part of the AEIOU intake procedure.

We will remain cognizant of the child's ability level and level of discomfort and distress at all times throughout the assessments. For example, prior to working with the children will we introduce ourselves to their parent(s) and ask for any insights regarding the child's behaviours and abilities. Using this information in combination with our own initial impressions of the child, we can select the module that seems developmentally/cognitively appropriate for the child. If, however, during the administration of the tasks the particular module is found to be too easy/difficult for the child, we will adjust the module selection accordingly. In addition, the parents are able to provide valuable information on how we can work with their child to minimise the distress/discomfort that they may experience. For example, when working with the child we can obtain advice on the appropriate distance to sit from the child (e.g. whether they like people to sit near or far away).

When coding and scoring the ADOS-2, it is useful to consult a video recording of the assessment. This supports discussion of coding amongst members of the research team. As such, parents will be asked to consent to their child being filmed while engaging in the ADOS-2 assessment. This video footage will only be available to the members of the research team and will be stored securely on the Griffith University server.

<u>Data Management</u>
Data will be controlled by Dr Adams who has extensive experience in managing Griffith's Living with Autism CRC-funded longitudinal study of students with autism (LASA). Data collection and management will be conducted in accordance with Griffith's schedule of retention periods for research data. All data will be de-identified. Each participant will be assigned an ID number. The key linking participant details and ID numbers will be stored separately from the data in a password-protected file. Publications produced from this project will only report anonymous combined results, not individual results.

All information collected and coded will be entered into password-protected files, only accessible by the project team. All media files (video files of the child-directed assessments and audio files of the intervention sessions) will be stored securely on the Griffith University server. Paper copies of questionnaires will be kept in locked filing cabinets separately from the consent forms. These will be stored in a locked cabinet in the researcher's office for at least 5 years from the point of data collection.

To further ensure security of data storage, the Griffith Research Storage platform will also be used (https://research-storage.griffith.edu.au/).

Data Analysis
All responses to the assessments will be scored as per the scoring guidelines allowing for quantitative analysis to conducted. This will determine if there is any change in pre/post intervention scores on the measures used in the project, and will allow for comparisons between the participant groups (intervention vs. control).

Data Linkage
N/A

Outcome measures (see attached questionnaire pack)
- Preschool Anxiety Scale Revised (PAS-R)
- Anxiety Scale for Children with Autism Spectrum Disorder (ASC-ASD)
- Intolerance of Uncertainty Scale (IUS-P)
- Vineland Adaptive Behaviour Scales 3rd Edition (VABS-3)*
- Repetitive Behaviour Scale – Early Childhood (RBS-EC)
- Depression Anxiety Stress Scales (DASS)
- Children's Anxiety Life Interference Scale (CALIS)
- Responses to Uncertainty and Low Environmental Structure questionnaire (RULES)
- Social Communication Questionnaire (SCQ; a measure of autism symptoms)
- Short Sensory Profile II (SSPII)
- School-related skills: These tasks are currently being developed and will be submitted as a modification prior to their implementation during 2021.

6. Results, Outcomes and Future Plans

Return of results/findings to participants.
The research team will write confidential reports for each parent summarising the results of the key assessments administered. These reports will be available after Stage 1 (for those recruited through social media), Stage 3 (short-term follow-up) and Stage 4 (one-year follow-up). The research team have extensive experience in assessment and reporting results. This report, together with participation in the intervention, will assist parents to learn more about anxiety and its management in relation to their child with autism.

All participating parents will also be invited to an end-of-trial celebration where we share the results with them.

Informing of future studies
The modified CLK program can potentially help many more parents of children with autism. This larger trial of the intervention will provide preliminary evidence of the effectiveness of the modified CLK program in the prevention/reduction of anxiety in autism.

Quality publication and dissemination of results
Publication of the intervention trial is planned, targeting a high-quality international peer reviewed journal. In addition, results will be presented at national and

international conferences. An article about the project will also be prepared and a lay summary shared through the Autism Centre of Excellence Facebook page and circulated through the participating early intervention centres. In all publications, presentations and articles, only de-identified group data will be used to ensure participant anonymity.

7. References

Carleton, R., Collimore, K., & Asmundson. (2010). “It’s not just the judgements - It’s that I don’t know”: Intolerance of uncertainty as a predictor of social anxiety. *Journal of Anxiety Disorders, 24*, 189-195. doi:10.1016/j.janxdis.2009.10.007

Craske, M. G., Kircanski, K., Zelikowsky, M., Mystkowski, J., Chowdhury, N., & Baker, A. (2008). Optimizing inhibitory learning during exposure therapy. *Behaviour Research and Therapy, 46*(1), 5-27. doi: 10.1016/j.brat.2007.10.003

Lawrence D., Johnson S., Hafekost J., Boterhoven De Haan K., Sawyer M., Ainley J., & Zubrick S.R. (2015). *The Mental Health of Children and Adolescents.* Report on the second Australian Child and Adolescent Survey of Mental Health and Wellbeing. Canberra: Department of Health.

Mychailyszyn, M. P. (2017). “Cool” Youth: A systematic review and comprehensive meta-analytic synthesis of data from the Cool Kids family of intervention programs. *Canadian Psychology, 58*(2), 105-115. doi: 10.1037/cap0000101

Rapee, R. M., Kennedy, S. J., Ingram, M., Edwards, S. L., & Sweeney, L. (2005). Prevention and early intervention of anxiety disorders in inhibited pre-school children. *Journal of Consulting & Clinical Psychology, 73,* 488-497. doi: 10.1037/0022-006X.73.3.488

Ung, D., Selles, R., Small, B. J., & Storch, E. A. (2015). A systematic review and meta-analysis of cognitive-behavioral therapy for anxiety in youth with high-functioning autism spectrum disorders. *Child Psychiatry and Human Development, 46*, 533-547. doi: 10.1007/s10578-014-0494-y

van Steensel, F., & Heeman, E. (2017). Anxiety levels in chidlren with autism spectrum disorder: A meta-analysis. J*ournal of Child and Family Studies.* doi:10.1007/s10826-017-0687-7

Vasa, R., Kalb, L., Mazurek, M., Kanne, S., Freedman, B., Keefer, A., Clemons, T. & Murray, D. (2013). Age-related differences in the prevalence and correlates of anxiety in youth with autism spectrum disorders. *Research in Autism Spectrum Disorders, 7,* 1358-1369. doi: 10.1016/j.rasd.2013.07.005
