# Supplementary material for: Protocol for a longitudinal study investigating the role of anxiety on academic outcomes in children on the autism spectrum
Source: PLoS One. 2021 Sep 16;16(9):e0257223. doi: 10.1371/journal.pone.0257223 (PMC8445440; doi:10.1371/journal.pone.0257223)
Supplement: S2 File — (PDF) [file pone.0257223.s003.pdf]

1. **Title**

"Cool for School": The impact of anxiety on the academic skills of children on the autism spectrum

2. **Project Team Roles & Responsibilities**

Dr Dawn Adams
Associate Professor, Autism Centre of Excellence, Griffith University.

Dr Adams is an associate professor in autism and a certified clinical psychologist with expertise in child and parental mental health. She has published extensively in the area of anxiety and autism over the past four years and is Lead CI on the ARC Linkage grant. Dr Adams will oversee the project and staff, the project budget, and coordinate the quantitative analysis and interpretation of the data, and management of the database for the project and reporting of results.

Dr Kate Simpson
Lecturer, Autism Centre of Excellence, Griffith University.

Dr Simpson is a lecturer in autism and a qualified occupational therapist and teacher. Her research focuses on how characteristics of autism influence participation and engagement across a range of environments. She will be responsible for training and oversight of the Research Assistant. She will contribute to data collection, analysis, and reporting of results.

Dr Stephanie Malone
Postdoctoral Research Fellow, Autism Centre of Excellence, Griffith University.

Dr Malone is a postdoctoral research fellow with experience in the development of children's early cognitive and academic skills. Dr Malone will have responsibility for the day-to-day management of the project to ensure efficient and effective coordination of activities. She will also assist with the recruitment of participants, participant assessments, recruitment and training of clinicians to implement the modified CLK program, data entry, analysis and reporting of results. She will also be the principal family liaison contact person and be responsible for ethical clearances and confidential storage of client records.

Dr Madonna Tucker
Research Manager, AEIOU Foundation.

Dr Tucker holds a key position within AEIOU Foundation, leading the organisation's program of research. She also has oversight of the child assessment and reporting program for the organisation. As such she has extensive experience liaising with families. For this project, she will be responsible for parent liaison, disseminating information to parents about recruitment, and answering questions from parents about the project.

Prof Ronald Rapee
ARC Laureate Fellow and Distinguished Profressor, Department of Psychology

Prof Rapee is a distinguished professor and ARC Laureate at Macquarie University. His research expertise centres on anxiety and related disorders across the lifespan, best known for theoretical models of development of anxiety disorders and creation of Cool Kids, Cool Kids for Autism and Cool

Little Kids. He will provide knowledge and expertise in the CLK program and guide modifications to the program with PI Rodgers and CI Adams. He will advise on training for intervention implementation, study design, and data analysis, and will contribute to reporting of results.

Dr Jacqui Rodgers
Senior Lecturer, University of Newcastle upon Tyne.

Dr Rodgers is a senior lecturer and autism researcher in the Institute of Neuroscience. She leads a programme of work that aims to advance the conceptualisation, assessment and treatment of anxiety in autism. Dr Rodgers will make modifications to the CLK program to incorporate material on intolerance of uncertainty to better target the CLK program for parents who have a child with autism.

3. Resources

This study is part of a two-phase nested project, funded by an ARC linkage project grant. The first phase (ethical approval number: 2019/989) focuses on working directly with children and their parents. The second phase – to which this application pertains – focuses on working with the teachers of children participating in the first phase. We appreciate that in order to gain the teachers' perspectives, it will be necessary for the children's parents to sign a second consent form (specifically relating to phase two). The parents, however, are under no obligation to provide permission for this second phase and are free to: a) continue with participation in the first phase only; or b) opt-in to participating in the second phase also. As always, parents are able to withdraw their participation from the research project at any time.

This study aims to capture teacher's perspectives on the academic performance of children on the autism spectrum. In order to obtain this, we seek ethical clearance from Brisbane Catholic Education, Education Queensland and necessary independent schools. Once permission is gained from their educational bodies, we will seek consent from the children's parents to gain the perspective of their child's teacher on academic performance. Consent will then be sought from the appropriate teachers. As always, participants are under no obligation to take part in this research.

Teacher directed assessments focus on gaining an understanding of the child's academic performance. This will be obtained using the Academic Competence Evaluation Scales – Teacher Form questionnaire. This questionnaire has 73 items in total; 33 relating to academic skills and 40 relating to academic enabler behaviours. It provides a measure of the child's performance in comparison to the grade expectations.

The funding source for this project is ARC LP180100318

4. Background

Research Program Structure: This study forms part of a nested program. The overarching aim is to investigate how anxiety, a commonly occurring condition in autism, impacts attitudes and behaviours that facilitate students' participation in, and

ability to benefit from, academic instruction in the classroom. The current study forms the **<u>second phase</u>** of this nested program.

As outlined in the successful Linkage Project grant application, this **first phase** program compares the anxiety levels of two groups of 4- to 5-year-old children with autism: those whose parents have completed the modified Cool Little Kids (CLK) intervention and those that have not. This will likely result in an overall group of children who have a range of anxiety levels; this is of interest in phase two (see below). The randomised control trial method used to allocate parents to the intervention and control groups will allow us to address a secondary aim: Is the modified CLK program able to reduce/prevent anxiety in children with autism? Although a larger scale RCT would be necessary to confidently demonstrate efficacy, the current study involving 64 families will provide important insights into the possible benefits of the intervention.

This ethics application seeks ethical approval from Griffith University for the **<u>second phase</u>** of this study. Additional ethical approval will also be sought from the necessary governing bodies (e.g. Brisbane Catholic Education, Education QLD). Parents who participated in phase one will be invited to take part in phase two. Data collected in phase two will include the teacher's perspective on the child's academic functioning (e.g. reading, mathematics and critical thinking) and academic enablers (e.g. motivation, engagement, interpersonal skills and study skills) within the year of formal schooling. By combining the data from phase one and two, we can determine whether moderating anxiety in children with autism is associated with academic enablers. This will allow our overarching research questions to be addressed:

1. Is there evidence that anxiety is associated with academic enablers in children with autism?
2. Does anxiety mediate the relationship between autism and academic enablers in children with autism?

Background Literature:

Children on the autism spectrum are consistently reported to underachieve academically compared to their ability (Ashburner et al., 2010; Mayes et al., 2007). These levels of underachievement remain present into adulthood (Kim et al., 2018), and are associated with reduced social and vocational outcomes (Magiati et al., 2014). Academic underachievement is understudied in autism, despite its high prevalence and potentially long-lasting impact. Consequently, little is known about associated factors (May et al., 2013). In a review of 19 studies investigating academic achievement in autism, Keen et al. (2015) identified no consistent predictors. Notable from the review was the lack of research on academic enablers: attitudes and behaviours that facilitate students' participation in and benefit from academic instruction in the classroom (DiPerna & Elliott, 2002).

Academic success relies on a combination of academic skills and enablers that include engagement, motivation, study skills, and social skills (DiPerna & Elliott, 2000). Our pilot research has shown that these enablers are poorer in students on the spectrum compared to children without autism. Critically, in children on the spectrum, these academic enablers were stronger predictors of academic achievement than language skills, even in children as young as five-years-old (Keen

et al., under review). It is not clear, however, why students on the spectrum have poorer scores on academic enablers, and whether it is possible to enhance these to improve academic outcomes. One plausible hypothesis is that anxiety (a common co-occurring experience in autism) is impacting on or possibly interacting with the child's academic enablers and their ability to engage and participate in education, making it more difficult for students to benefit from academic instruction in the classroom and contributing to poorer academic achievement.

Anxiety disorders are the most common form of mental disorder in young people, affecting around 7% of Australian youth (Lawrence et al., 2015). These disorders are even more common in children with autism, affecting up to 40% of the population (van Steensel & Heeman, 2017) with an even larger number showing sub-clinical levels of elevated anxiety (Vasa et al., 2013).

Among typically developing children, anxiety is a strong predictor of poor school engagement and performance (Wood, 2016). Anxiety has been linked to below-grade-level academic achievement, school failure, and academic skill impairment (Nail et al., 2015). Subsequently, reducing anxiety has led to improved school engagement and performance (Wood, 2016). Based on these relations, the dramatic impact of autism on academic achievement may be at least partly a consequence of the high levels of associated anxiety.

Given that both academic outcomes and academic enablers are poorer in children on the spectrum, it is reasonable to hypothesise that the poor academic success noted within this population is likely a consequence of the impact of autism on the development and/or implementation of academic enablers. It is also highly likely that anxiety is impacting on these same enablers. For example, extensive evidence with older, typically developing children has demonstrated the impact of anxiety on social performance and interactions (Greco & Morris, 2005). Similarly, perfectionism and worry which are key components of anxiety, can have a negative impact on study skills and motivation. At a broader level, enablers of good academic functioning often reflect the ability to focus attention and think flexibly. A core mechanism of anxiety, threat expectancy, interferes with a range of cognitive processes, including attentional focus (Cisler, 2010).

In summary, it is likely that heightened anxiety interacts with academic enablers to contribute to the poor academic performance consistently reported for children on the spectrum. However, to date, this hypothesis has not been tested.

5. Project Description

Setting

Teachers will be asked to complete the ACES-TF using an online questionnaire via REDCap. They are able to complete this questionnaire in a location that they feel comfortable (e.g., at school or at home).

Methodological Approach

This is a nested program of research; those parents and children recruited during Phase One (approval number: 2019/989) will be invited to participate in Phase Two. Parents and children on the autism spectrum (aged 4- to 5-years-old) were recruited

to Phase One via social media (Griffith University Autism Centre of Excellence Facebook page), early intervention centres operated by our research partner (AEIOU Foundation) and other community groups. In order to gain information about the children's academic performance, we will approach the teachers of these same children. Given that the children are located throughout SE Queensland (i.e., Logan to Caboolture), it is likely that they will attend a diverse range of schools meaning that it is unlikely that each Preparatory Year teacher will work with multiple children within our sample.

The collection of the teacher data will coincide with the final time point (Stage 4) of Phase One. This will occur during the academic year 2021 (for children recruited to Phase One in 2020) and 2022 (for children recruited to Phase One in 2021).

<u>Participant Recruitment</u>

Parents participating in Phase One will be approached via email which will provide information on Phase Two. It will also outline how this information will be used to supplement the parent- and child-data collected from Phase One, while emphasising that they are under no obligation to participate in Phase Two. The information sheet and consent form will be attached to this email, as well as providing a link to an online information sheet and consent form (via REDCap). Parents can opt to provide consent via the link or by returning a signed copy of the consent form. If they choose to participate in Phase Two (i.e., give consent for the researcher(s) to approach their child's Preparatory Year teacher), they will be asked to provide the teacher's name and school.

School: Once parental consent has been obtained, their child's school will be approached first via telephone to obtain the most appropriate contact details (i.e., head of school). An email will then be sent to the main contact to obtain permission for the researcher(s) to contact the relevant teacher regarding their participation in the research project.

Teacher: An email will be sent to the teacher using the contact details provided by the school's main contact. This email will explain that a child in their class is part of an ongoing research project, as well as providing information about the teacher questionnaire. The teacher information sheet and consent form will be attached to this email, along with a link to the online questionnaire (hosted via REDCap).

Please note, no contact will be made with the school or teacher until ethical approval has been obtained from the relevant governing body (i.e., EdQLD and BCE).

<u>Participants</u>
This is part of a nested project. The same parents and children (4- to 5-years-old at recruitment) recruited to Phase One (approval number: 2019/989) will be approached to participate in the current Phase (Phase Two). Once parental consent is obtained, the child's school and teacher will be approached. Given that our participating children come from diverse geographically locations around South East Queensland, it is unlikely that a single teacher will have multiple children from our cohort in their class. As such, we hope to have approximately 64 teacher's providing information about the children's early school abilities.

Recruitment strategy:

Email to parents: An email will be sent to parents participating in Phase One of the research project. This email will invite these parents and children to participate in the second phase of the project: teacher perspectives on child's academic abilities. Parents will be asked to provide consent by completing the online form provided on REDCap.

Email to school: An email will be sent to the main contact at the child's school, requesting permission for the researcher(s) to contact the child's Preparatory Year in order to provide information about the research project.

Email to teacher: An email will be sent to the child's teacher via their school email account. This email will explain that a child in their class is participating in an ongoing research project examining the link between anxiety and school performance. Teachers will then be invited to complete an online survey (hosted by REDCap). The survey will begin with an online information sheet and consent form, before progressing to the ACES-TF questionnaire.

Incentive: Teachers will be provided with a $100 gift card after their completion of the online survey. In the unlikely event that a single teacher has multiple participating children in their class, they will receive a gift card to the same value for each survey they complete.

Procedure after consent is given

Upon obtaining consent, one of our researchers will contact consenting parents and provide information about Phase Two of the project. Upon obtaining content (via REDCap or return of a signed consent form), their child's school and teacher will be contacted, as described above. Teachers will be provided with a link to the online information sheet, consent form, and questionnaire. After completion of the questionnaire, teachers will be sent a gift card in the post as a thank-you for their participation in the research. Teachers, however, are free to opt-out of the research project.

Teacher data will then be matched with the parent- and child-data collected during Phase One of the project.

Research Activities

Teacher's will be provided with a link to the ACES-TF questionnaire. This is a 73-item questionnaire examining children's performance in academic skills (e.g., reading) and academic enablers (e.g., motivation). Given the length of this questionnaire, it is expected that it will take approximately 40 minutes to complete.

Data Collection/Gathering

Parents: All parents will be asked to provide the name of the school in which their child is completing their Preparatory Year and the name of their child's main teacher.

Teachers: Teachers will be asked to complete the ACES-TF questionnaire assessing children's academic performance. This will be completed using REDCap – a secure online, survey tool.

<u>Data Management</u>
Data will be controlled by Dr Adams who has extensive experience in managing Griffith's Living with Autism CRC-funded longitudinal study of students with autism (LASA). Data collection and management will be conducted in accordance with Griffith's schedule of retention periods for research data. All data will be de-identified. Each teacher will be assigned an ID number. The key linking teacher details (and therefore their responses to the questionnaire) and ID numbers will be stored separately from the data in a password-protected file. Publications produced from this project will only report anonymous combined results, not individual results.

All information collected will be saved securely on the REDCap server. Any data that is downloaded will be de-identified and saved on a password protected server at Griffith University, accessible only by the members of the research team. Paper copies of returned consent forms will be kept in locked filing cabinets separately from any associated data (e.g., child's responses from Phase One). These will be stored in a locked cabinet in the researcher's office for at least 5 years from the point of data collection. **To further ensure security of data storage, the Griffith Research Storage platform will also be used (https://research-storage.griffith.edu.au/).**

<u>Data Analysis</u>
All responses to the assessments will be scored as per the scoring guidelines allowing for quantitative analysis to conducted. This will determine if there is any relation between anxiety and school performance.

<u>Data Linkage</u>
This study is part of a nested project. The teacher data obtained during this phase will be linked with the parent- and child-data obtained in Phase One. The key linking teacher responses with child- and parent-responses will be saved in a password protected file, accessible only to researchers listed on this application.

<u>Outcome measures (see attached questionnaire pack)</u>

- Academic Competence Evaluation Scales – Teacher Form

6. Results, Outcomes and Future Plans

<u>Return of results/findings to participants.</u>
The research team will provide a summary report on the findings of the project. This information will be circulated to participating parents and teachers, which will assist parents and teachers to learn more about anxiety and its relation to academic performance in children on the autism spectrum.

<u>Informing of future studies</u>

This research can will help to identify the potential link between anxiety and academic performance in school, which can ultimately inform the development of interventions to assist in supporting children on the autism spectrum at school.

Quality publication and dissemination of results
Publication of the research is planned, targeting a high-quality international peer reviewed journal. In addition, results will be presented at national and international conferences. An article about the project will also be prepared and a lay summary shared through the Autism Centre of Excellence Facebook page and circulated through the participating early intervention centres. In all publications, presentations and articles, only de-identified group data will be used to ensure participant anonymity.

7. References

Ashburner, J., Ziviani, J., & Rodger, S., *Surviving in the mainstream: Capacity of children with autism spectrum disorders to perform academically and regulate their emotions and behavior at school.* Research in Autism Spectrum Disorders, 2010. **4**(1): p. 18-27.

DiPerna, J.C., & Elliott, S. N., *Academic Competence Evaluation Scales (ACES).* 2000, San Antonio, TX: Psychological Corporation.

DiPerna, J.C., & Elliott, S. N., *Promoting academic enablers to improve student achievement: An introduction to the miniseries.* School Psychology Review, 2002. **31**: p. 293-297.

Greco, L.A., & Morris, T. L., *Factors influencing the link between social anxiety and peer acceptance: Contributions of social skills and close friendships during middle childhood.* Behavior Therapy, 2005. **36**: p. 197-205.

J.M. Cisler, E.H.W.K., *Mechanisms of attentional biases towards threat in anxiety disorders: An integrative review.* Clinical Psychology Review, 2010. **30**: p. 203-216.

Keen, D., Adams, D., & Simpson, K., *Academic skills and enablers in a community sample of children with autism.* under review.

Keen, D., Webster, A. & Ridley, G. , *How well are children with ASD doing academically at school? A review of the literature. .* Autism: The International Journal of Research and Practice, 2015. **20**: p. 276-294.

Kim, S., Bal, V., & Lord, C., *Longitudinal follow-up of academic achievement in children with autism from age 2 to 18.* Journal of Child Psychology and Psychiatry, 2018.

Lawrence D., J.S., Hafekost J., Boterhoven De Haan K., Sawyer M., Ainley J., & Zubrick S.R., *The Mental Health of Children and Adolescents. Report on the second Australian Child and Adolescent Survey of Mental Health and Wellbeing*, D.o. Health, Editor. 2015: Canberra.

Magiati, I., Tay, W. X., & Howlin, P., *Cognitive, language, social and behavioural outcomes in adults with autism spectrum disorders: A systematic review of longitudinal follow-up studies in adulthood.* Clinical Psychology Review, 2014. **34**: p. 73-86.

May, T., Rinehart, N., Wilding, J., & Cornish, K., *The role of attention in the academic attainment of children with Autism Spectrum Disorder.* Journal of Autism and Developmental Disorders, 2013. **43**: p. 2147-2158.

Mayes, S., & Calhoun, S., *Learning, attention, writing, and processing speed in typical children and children with ADHD, autism, anxiety, depression, and oppositional-defiant disorder.* Child Neuropsychology, 2007. **13**: p. 469-493.

Nail, J., Christofferson, J., Drake, K., Kendall, P., McCracken, J., Birmaher, B., . . . Sakolsky, D., *Academic impairment and impact of treatments among youth with anxiety disorders.* Child and Youth Care Forum, 2015. **44**: p. 327-342.

van Steensel, F., & Heeman, E., *Anxiety levels in chidlren with autism spectrum disorder: A meta-analysis.* Journal of Child and Family Studies, 2017.

Vasa, R., Kalb, L., Mazurek, M., Kanne, S., Freedman, B., Keefer, A., Clemons, T. & Murray, D., *Age-related differences in the prevalence and correlates of anxiety in youth with autism spectrum disorders.* Research in Autism Spectrum Disorders, 2013. **7**: p. 1358-1369.

Wood, J., *Effect of anxiety reduction on children's school perfromance and social adjustment.* Developmental Psychology, 2006. **42**: p. 345-349.
