# Supplementary material for: Protocol for a longitudinal study investigating the role of anxiety on academic outcomes in children on the autism spectrum
Source: PLoS One. 2021 Sep 16;16(9):e0257223. doi: 10.1371/journal.pone.0257223 (PMC8445440; doi:10.1371/journal.pone.0257223)
Supplement: Attachment — Submitted filename: Response to reviewer 27.5.21.docx [file pone.0257223.s004.docx]

We thank the editor and the reviewers for their feedback on the manuscript. We have attended to each reviewer’s comments in turn below. Changes to the manuscript are marked in blue text.

| Journal requirements |  |
| --- | --- |
| Please ensure that your manuscript meets PLOS ONE's style requirements, including those for file naming. Please include the clinical trial registration information in your Methods section | This has been added to the methods section. |
| Update competing interests into cover letter | This has been updated in the cover letter |
| Data availability statement | This has been updated to state that the manuscript does not have data and the data availability policy is therefore not applicable to this article. It has also been updated to state that the anonymised dataset for the findings of the study outline in the Registered Report Protocol will be stored in Griffith’s Research Data Repository, which is accessible and searchable through a web interface. <https://www.griffith.edu.au/library/research-publishing/repository> |
| 5. Your ethics statement should only appear in the Methods section of your manuscript. If your ethics statement is written in any section besides the Methods, please delete it from any other section. | The ethical information has now been removed from the abstract. |
| 6. Please include captions for your Supporting Information files at the end of your manuscript, and update any in-text citations to match accordingly. | Caption for Figure 1 has been added into the document. |
| Comments to the author – Reviewer 1 |  |
| Reviewer #1: I appreciate this opportunity to review this nicely planned research proposal. I would like to apologize for the late reply. I have some comments and suggestions before fully endorsing the publication of this proposal. | We thank the reviewer for this positive feedback. |
| 1) On Page 6, the authors directly stated “One plausible hypothesis is that anxiety….” I think the publication of this proposal is also of educational purpose, it thus is worth a short mentioning of other potential contributors, e.g. executive dysfunction and motivation problem, etc. | We have added in a statement to highlight the possible role of EF. In a review of 19 studies investigating academic achievement in autism, Keen *et al*. identified no consistent predictors.[7] Since this was published, there have been some studies linking previously unexplored factors, such as some elements of executive functioning, to academic attainment.  As Motivation is listed as an example of an academic enablers, this already discussed in the introduction. |
| 2) On Page 8, “In summary, it is likely that heightened anxiety…..” I appreciate the novel endeavor to link anxiety and academic enabler issues associated with children with ASD. However, there still are numerous other contributing factors to the problems in academic enablers, as mentioned in the last points. Therefore, I suggest that the summary sentences of Introduction should be toned down a bit to something like, “it is likely that heightened anxiety, at least partially, interacts with….” | We have made the suggested amendment, so the sentence now begins “In summary, it is likely that heightened anxiety, at least partially, interacts with academic enablers” |
| 3) On Page 9, Aim and hypotheses: I find that the modified CLK actually is based on the CLK and further integrates the element of CUES, as explained in the preceding paragraph. However, I don’t find the reason why only the CLK is highlighted in the description of the distinct intervention herein. | Thank you, we have amended the manuscript to make it clear for the reader that it is the modified CLK and CUES interventions, and now refer to it as CLK-CUES. |
| 4) On Page 10, as the Preparatory Year should be a very Aussie-specific system, it is worth a concise introduction of this system to the non-Aussie readers. | We have removed this term and put “first year of formal schooling” so that it is clear for readers of all nationalities. |
| 5) Would the interindividual differences in early intervention impact the results? This element seems not to be taken into account in the data analysis part. Similarly, the interindividual differences in receiving services assisting with anxiety are not accounted for in the data analysis model. | The randomisation design limits the impact of confounding factors such as early intervention, as these will be randomly distributed between the two groups and therefore randomly distributed across those with higher and lower anxiety. However, to ensure we are able to explore this factor on outcomes, we have added in a statement at the start of the measures section to clarify that the parent questionnaire at Time 2 and 3 includes questions on help, therapy or medication received for anxiety since the last assessment. Data screening will identify any relationship between this and other variables. |
| 6) It seems that this study does not exclude autistic children with co-occurring intellectual impairments. I think this practice is highly laudable. However, I wonder whether intellectual levels would impact the features of academic enablers, and the assigned school options, where the teachers might estimate the academic enablers differently, considering the different expectations for those with co-occurring intellectual disability. | Thank you for acknowledging the importance of including young children with intellectual disabilities within the trial, as I’m sure you are aware, children on the autism spectrum with co-occurring ID make up only 6% of participants in trials reviewed by Russell et al. (2019). We are therefore very motivated to include participants of all abilities in this study.  As noted above, the randomisation approach will ensure that confounding variables are equally distributed across the control and experimental group, and therefore hypothetically equally distributed across the spread of anxiety levels.  The ACES asks teachers to rate the student in comparison to their peers. We have previously explored the ACES in a sample of 113 children on the autism spectrum. We did not see anything which would suggest a rating difference due to expectations, for example, for all ACE subscales, the children in special education settings were rated as lower than those in mainstream inclusive settings. This gives us confidence that teachers are rating as required by the ACES, which is comparison to other children of the same age. However, in our data screening section, we can check for any impact of teacher ratings for those with and without a co-occurring ID. |
| 7) On Page 13, the authors explicitly stated that this trial should not be considered a clinical trial. To me, this statement is inaccurate, as the secondary outcome actually involves the changes of anxiety after the CLK+CUES. This statement also contradicts with the discussion in the following paragraph, in which the potential significance in clinical improvement from the intervention is highlighted. Furthermore, as the authors highlighted, there has yet to be data on the CLK or CUES efficacy on anxiety in autism. The results of the current proposal certainly could generate the pilot data for the next-stage clinical trial. | We appreciate that this may have been confusing for the reader so we have removed this statement. |
| Reviewer 2 |  |
| The protocol clearly describes an important research question, it is well written and seems to address all the key issues. However, my main concern, see also some specific points below, is the proposed complex and multi-faceted analysis described (Page 19). Further I suspect the sample size eventually achieved may not be sufficient to justify the analytical methods proposed. My general suggestion is therefore to simplify the protocol as much as possible and thereby reduce the amount of data collected so as to focus on key questions. Otherwise, I fear that there is a danger that the trial will fail for logistical reasons (too much missing data) and the key questions may not be answered. In brief, overloading the parents/children and the investigating team will diminish the chance of a successful completion of this randomised trial. | Thank you for this positive feedback. We appreciate that the parents have a large questionnaire pack to complete at each time point. To minimise the risk of missing data, we ensure that parents are asked to complete the primary outcome variables first, before moving on to the additional questionnaires which will only form part of secondary exploratory analyses (informed by the initial findings). We will also use this approach for the teacher questionnaire. |
| As this is a ‘randomised trial’ I suggest this term replaces ‘study’ in the title. | Whilst this study does use a randomised trial to experimentally manipulate the levels of anxiety across the sample, the primary aim is looking at how anxiety impacts academic outcomes (not whether the intervention proves effective in a randomised trial). We have tried to make this clearer throughout for our reader. We would therefore like to have the title of “Protocol for a longitudinal study investigating the role of anxiety on academic outcomes in children on the autism spectrum” so that the reader does not come into the protocol assuming a trial of an intervention, but a study which uses experimental manipulation of anxiety to explore the impact of anxiety on academic outcomes. |
| Endpoints (Pages 9 and 10): The aims of the trial are not expressed as evaluating the effectiveness of the Anxiety Reduction/Prevention program (ARP) (a combination of CLK and CLUES) as compared to a non-intervention Control at 2-years post baseline (T3). Although this anticipated difference does form the basis of the sample size calculation (Page 11). Neither do they clearly state the aim in relation to the assessment at time T2. | Thank you for highlighting this discrepancy. The only way to infer causation is through experimental manipulation. The intervention is being used as a method to experimentally manipulate anxiety across the entire sample, so that causation on academic outcomes can then be inferred. The case for needing to experimentally manipulate anxiety levels is strengthened by the published research which suggests that 70-80% of children on the autism spectrum experience elevated anxiety (discussed in the introduction). Without an intervention to reduce anxiety levels in some of the participants, there is a risk that there will not be enough participants with the lower anxiety scores to explore the impact of a range of anxiety levels on academic outcome. The randomisation to intervention/control group was done to minimise potential bias in confounding variables between those who did and did not receive the intervention, i.e. those who should have lower or higher levels of anxiety.  The primary analysis is therefore focused upon the academic outcomes and the measure of anxiety. However, it is prudent to check if the experimental manipulation worked, hence the T2 assessments. This experimental manipulation check will form part of initial data screening and participant description section.  Based on your helpful feedback relating to the effect size, we have updated this to reflect the need to (a) ensure enough participants to see differing levels of anxiety between the two groups and then (b) enough participants to conduct the linear regression exploring the impact of anxiety (and its possible moderating effect) on academic outcomes. |
| Recruitment and Randomisation (Page 10): The protocol seems to suggest that there may be considerable delay between randomisation and the commencement of the intervention. In general terms, to avoid losses (parents/children changing their minds about participation) it is best to start the intervention (whether ARP or Control) as soon as possible after randomisation. Similarly baseline values should be recorded immediately prior to the randomisation. However, I am not sure of the logistical problems here. | Upon re-reading, the sentence was misleading in suggestion a considerable delay. We have amended it to clarify the process as per below  Groups will be run during school-terms to reduce any barriers relating to childcare. In order to minimise the risk of participant drop out between randomisation and commencement of the intervention, recruitment advertising will be timed to enable recruitment, initial assessment and randomisation occurs as close to the onset of the groups as possible whilst also allowing parents sufficient notice of group onset. |
| 4. Randomisation (Page 12): More detail of the randomisation process is required. This includes: Who generates the list (with details of block size). Also, who holds the list (someone independent of the investigators) and how is the randomisation carried out (by phone to as statistical office?). | Additional information has been added to page 12 and 13 to clarify the randomisation process.  “For each wave, the project manager - who is independent to the investigators - will use a random number generator, to randomly allocate…” (p. 12)  “This project manager will also maintain a record of participants allocated to the conditions” (p. 13) |
| 5. Sample size (Page 10): For the primary endpoint of the Academic Competence Evaluation Scales (ACES) at 2-years (Is this correct?) post-randomisation the standardised effect size, d = 0.75, then with test size 5%, power 80%, attrition rate (10%) I can confirm the sample size of N = 64 children. However, it would be useful if a direct (statistical) reference is given to the calculation method utilised.  6. Primary analysis: The anticipated attrition implies that only 58 children may be available for analysis. Although this is sufficient for the planned t-test (the phrase ‘and non-centrality parameter’ is not relevant here), I suggest that some of the further analyses, such as hierarchical multiple regression (page 11), will not be very sensible with such numbers. Neither does this method seem relevant to answering the main research question (see Point 2 above). This analysis requires the estimate of a difference between two means via the t-test, with the corresponding confidence interval. In addition, linear regression including intervention and the baseline assessment ACES may be useful.  7. Secondary analysis (page 19, last paragraph): Surely all that is needed here is a similar approach to Point 5 above. I don’t anticipate ANOVA would be required. | Thank you for confirming the calculation is correct for that part of the power analysis. We have also added a reference to G power. Based on your feedback, we have expanded our power analyses section to reflect the need to (a) ensure enough participants to see differing levels of anxiety between the two groups and then (b) enough participants to conduct the linear regression exploring the impact of anxiety (and its possible moderating effect) on academic outcomes. We have also clarified the analysis plan, and incorporated the moderation within a linear regression rather than through PROCESS. |
| Reviewer 3 |  |
| Strengths: This type of study examining the role of anxiety on the academic performance and learning in autistic children is much needed as they may pave the way to the successful development of school-based intervention that can improve outcomes in autistic children.  Use of parent- and teacher-rated measures, use of multiple measures including sensory, behaviour-related, cognitive and adaptive scales.  Carefully designed intervention, taking into consideration some common potential pitfalls.  Having an author from the population health sciences institute. | Thank you for recognising these strengths in the study. |
| In my opinion, there are several limitations of this protocol:  I am not clear about the exact primary measure of this protocol. In the abstract and data analysis section Academic Competence Evaluation Scales - teacher rated form was mentioned as primary but in the table Wechsler individual achievement test was mentioned as the primary. | We apologise for this being unclear. The primary outcomes are the academics – the teacher-rated Academic Competence Evaluation Scales and direct assessment of the WIAT-II. Having both of these as measures of academic outcomes minimises the risk of attrition through teacher non-completion of questionnaires. |
| It would be easier to interpret if authors could add a figure to describe the study design. | A figure has been added to clearly describe the study design |
| Authors mentioned that there was no identified predictor of academic underperformance in autistic children. They reviewed literature to support their focus on anxiety in autistic children. However, this link appears non-specific as they also mentioned that anxiety is a strong predictor of academic underperformance in neurotypical children as well. Furthermore, given that clinically manifested anxiety is seen in 4 out of 10 autistic children, how would they justify the distribution of anxiety between the two groups? Would there be any value in having a neurotypical control group with anxiety? How about autistic children with and without significant anxiety to control for non-anxiety related autism factors contributing? | We have revised the manuscript to try to ensure the aim is clear to the reader.  The primary aim is to explore whether anxiety impacts academic outcomes for young children on the autism spectrum and whether it moderates the relationship between autism and academic outcomes. Given the notable difference in experience and presentation of anxiety in autism compared to neurotypical children, and the differing effect sizes of established therapies (e.g. CBT) it is important to conduct autism-specific research.  We agree with you that there needs to be a range of anxiety levels across the participant groups. This is the reason that we have designed a study that aims to experimentally manipulate the level of anxiety (i.e. lower the anxiety) in some of the sample. The intervention is not the focus of this study (the focus is on the impact of anxiety on academic outcomes), the intervention is just a way to infer causation between anxiety and the primary outcome (academic outcomes, enablers). The randomisation to intervention/control group was done to minimise potential bias in confounding variables between those who did and did not receive the intervention, i.e. those who should have lower or higher levels of anxiety. |
| The authors included potential moderators (e.g. parental mental health, etc) in the analysis but they did not review information on these potential moderators related to autism in the introduction. | On the basis of the advice from the statistical advisor (reviewer 2), this has been removed. |
| There are no exclusion criteria - what about unstable or uncontrolled medical conditions interfering with the academic ability, eg, epilepsy (common in autistic children), specific learning disability, intellectual disability, speech problem, genetic disorders that affect mobility and communication, presence of co-occuring mental health conditions such as attention-deficit hyperactivity disorder that affects academic performance. The design, if focused on anxiety as a mediator in the context of ‘autism’, should consider all these potential confounders. | The randomisation design limits the impact of confounding factors such as medical conditions, genetic disorders etc. should be randomly distributed between the two groups and therefore distributed across the spread of anxiety levels. We have noted that future work with larger samples could account for all of these additional factors at the end of the manuscript as per below.  “Such larger trials could also then account for other co-occurring conditions with may impact upon academic outcomes and/or interact with anxiety in children on the autism spectrum.“ |
| Authors mentioned effect size calculation to justify the sample size but it is not clear if they referred to Cohen’s d and why did they use t-statistic when the proposed analysis plan includes multiple hierarchical regression, mixed ANOVAs. | Based on the feedback from the statistical advisor (reviewer 2) we have clarified the effect size calculations on page 13. |
| The process of blinding is not also clear to me, for instance, authors mentioned that the research team members collecting data will be blind. However, the CLK intervention will be administered by a trained member of the research team and a second research team member will assess the validity. A separate team of analysts/researchers need to deliver the intervention. Also, will the data analysts be blind? | This has been clarified on page 14.  The research team consists of   1) project manager, who focuses upon recruitment, arrangement of assessments, database management and contact with participants. As they cannot be blind (as they send the parents the group information, speak to parents etc.) they cannot be blind. 2) Research assistant who conducts and scores the direct assessments with the children and collects the parent questionnaires. They are blind to the condition. 3) The clinician who delivers the intervention. They are obviously not blind but do not collect any data. 4) The remaining four research team members are not involved with the day-to-day data collection or parent groups. They are blind to all identifying participant information and conditions. One of these team members will assess the validity of 20% of the group sessions, blind to participant information.   The data analyst will be blind to any identifying information on participants (data will be entered by ID only) but as part of data screening is to ensure the experimental manipulation of anxiety worked, there will be a variable in the dataset which identifies if the participant was in the experimental or the control group. They therefore cannot be blind to the experimental group. |
| In the data analysis plan, authors need to include details regarding controlling for multiple comparisons. The sample size should reflect that as well but a sample size of 64 is not sufficiently justified. | The statistical advisor (reviewer 2) has verified the sample size calculation. |
| The authors will collect information on sensory profiles, adaptive, etc but how they are going to handle all these additional measures in the analysis plan? It remains unclear.  I did not see any sex/gender consideration, which is a potentially powerful mediator in autism. | These will be explored in secondary exploratory analyses, informed by the outcomes of the primary analyses.  There is no consensus as to whether anxiety differs for males and females on the spectrum (see literature review in Ambrose, Adams, Simpson & Keen, 2020). However, this could be explored in the exploratory analysis if data suggests this to be a useful analysis. |
| Reviewer 4 |  |
| Thank you for the opportunity for reviewing this manuscript. This is a research protocol investigating the relation between anxiety and academic achievement in children on the autism spectrum. This randomized longitudinal study aims to study the moderating effects of anxiety in development of academic enablers in children with autism spectrum disorder. Secondary outcome measures of the study are academic attainment and anxiety. The proposed study has a randomised, longitudinal design and a sample size of 64, based on appropriate sample size calculation. A battery of parent-reported measures and academic performance measures are proposed to test the hypothesis. The authors have used a robust methodology for this study and have addressed issues related to recruitment bias and waitlist control in the study. Use of SPIRIT checklist add rigour to the quality and transparency of the study protocol. This methodologically sound study can contribute to current understanding of the role of anxiety in autism and has the potential to inform development of interventions in children with autism in their early school years. The authors of the study protocol clearly state the rationale for the study with clearly defined research questions. They also state that the hypothesis of heightened anxiety interacting with academic enablers to contribute to poor academic performance of children on the spectrum has not been tested to date. In this study, a modified anxiety reduction/prevention program comprising of Cool Kids program (CLK), a parent-led program for anxiety in pre-school children and Coping with Uncertainty in Everyday Situations (CUES), another parent mediated intervention, will be used as an intervention for anxiety. | Thank you for this positive feedback and for providing your review. We have addressed your comments below and amended the manuscript as required. |
| The authors have mentioned about a pilot study on the role of CLK in anxiety intervention. Please describe the modified program and cite additional supporting references, if any, about the role of CLK in moderating anxiety in autism. | The impact of the standard CLK program on anxiety outcomes of preschoolers on the autism spectrum is described on page 9 “In a recent analysis, Bischof *et al* showed that CLK also led to reductions in anxiety among a group of preschool-aged children with comorbid autism, with feedback from parents suggesting that future offerings of the intervention could use examples and information that was more autism focused.[33]”  The pilot of the CLK-CUES program was only on three children and focussed on acceptability of the intervention rather than anxiety outcomes. |
| CLK has been used for children in the age group 8-15 years and this study is designed for 4–5-year-old children on the spectrum.  A pilot study of the modified version has been carried out but it is not clear if the participants were pre-school children | The team at Macquarie have developed multiple parent interventions; Cool Kids (for children aged 7-17) and Cool Little Kids (for children 3-6 years old). We have made this distinction clearer in our protocol.  For clarity, this study is looking at a modified version of Cool Little Kids, so the intervention we are proposing is the one for the correct age range. We have also clarified that the pilot study was on parents of pre-school children on the autism spectrum. |
| In addition, the validity of the modified anxiety reduction program as an intervention for the participants is not clear. Please address this issue in the research protocol. | The primary aim of the study is to explore whether anxiety impacts academic skills and outcomes for children on the autism spectrum after they transition into school. To ensure a range of anxiety levels, the CLK-CUES intervention will be used for some, but not all children.  We have added in additional information on page 13 to make it clear that CLK-CUES intervention contains all of the CLK elements but then has additional information added in.  As the CLK intervention has shown significant effect at reducing anxiety in young children with autism (Bischof et al. described above), there is no reasonable reason to believe that adding in additional information to the CLK program to make it autism specific will make it less effective. |
